# Molecular characterization of a polymycovirus in *Leptosphaeria biglobosa*

**DOI:** 10.1007/s00705-025-06253-1

**Published:** 2025-03-06

**Authors:** Nesma M. Samir, Jacob Locke-Gotel, Syun-Ichi Urayama, Adel A. El-Morsi, Ghada A. El-Sherbeny, Yongju Huang, Bruce D. L. Fitt, Hiromitsu Moriyama, Robert H. A. Coutts, Ioly Kotta-Loizou

**Affiliations:** 1 Laboratory of Molecular and Cellular Biology, Department of Applied Biological Sciences, Tokyo University of Agriculture and Technology, 3-5-8, Saiwaicho, Fuchu, Tokyo 183-8509 Japan; 2 Department of Clinical, Pharmaceutical and Biological Science, School of Life and Medical Sciences, University of Hertfordshire, Hatfield, AL10 9AB UK; 3 Laboratory of Fungal Interaction and Molecular Biology (donated by IFO), Department of Life and Environmental Sciences, University of Tsukuba, 1-1-1 Tennodai, Tsukuba, Ibaraki 305-8577 Japan; 4 Department of Botany, Faculty of science, Mansoura University, Mansoura, 35516 Egypt; 5 Department of Life Sciences, Faculty of Natural Sciences, Imperial College London, London, SW7 2AZ UK

## Abstract

*Leptosphaeria biglobosa* is a phytopathogenic ascomycete of *Brassica napus* that causes phoma stem canker/blackleg. A new double-stranded RNA (dsRNA) mycovirus from this fungus has been fully characterized. The virus genome has five dsRNA segments, ranging in length from 1,180 bp to 2,402 bp. Each dsRNA has a single open reading frame flanked by 5′ and 3′ untranslated regions. The proteins encoded by dsRNAs 1 and 3, an RNA-dependent RNA polymerase (RdRP) and a methyltransferase, respectively, have significant similarity to those of Plasmopara viticola lesion associated polymycovirus 1. The proline-alanine-serine-rich protein encoded by dsRNA 5 is similar to that of Erysiphe necator associated polymycovirus 1. The proteins encoded by dsRNAs 2 and 4 have significant similarity to those of a mycovirus identified in *Alternaria* sp. FA0703. Phylogenetic analysis based on RdRP sequences showed that this virus clusters with members of the family *Polymycoviridae*. Based on these observations, this virus, which we have named "Leptosphaeria biglobosa polymycovirus 1", should be classified as a member of the family *Polymycoviridae*. This is the first report of a polymycovirus in* L. biglobosa.*

**Supplementary Information:**

The online version contains supplementary material available at 10.1007/s00705-025-06253-1.

Viruses infect all forms of cellular life, and fungi are no exception. Knowledge regarding the diversity of fungal viruses, or mycoviruses, is growing rapidly [1]. The majority of mycoviruses have a double-stranded (ds) RNA or positive-sense (+) single-stranded (ss) RNA genome [1]. There are more than 30 established mycovirus families, many of which include mycoviruses with multi-segmented dsRNA genomes: e.g., the families *Alternaviridae* (4 segments) [2–4], *Chrysoviridae* (3-7 segments), *Quadriviridae* (4 segments)*,* and *Polymycoviridae* (4-9 segments) [5]. All polymycoviruses reported thus far have four conserved genome segments, each with an open reading frame (ORF) flanked by 5′ and 3′ untranslated regions (UTRs). These ORFs encode an RNA-dependent RNA polymerase (RdRP), a protein of unknown function, a methyltransferase, and an intrinsically disordered proline-alanine-serine-rich protein (PASrp) [4, 6–12]. Several polymycoviruses also have up to five additional genome segments containing ORFs that encode proteins of unknown function [4, 6–8, 13].

Phoma stem canker or blackleg is a serious disease that affects *Brassica napus* (commonly known as oilseed rape or canola) worldwide and results in annual yield losses to the global rapeseed industry of up to 160 million US dollars [14]. Phoma stem canker pathogen populations are made up of two main species: *Leptosphaeria biglobosa*, which is linked to less-harmful upper stem lesions, and *Leptosphaeria maculans*, which is linked to devastating stem base cankers [15]. Several dsRNA mycoviruses, including those belonging to the genus *Botybirnavirus* [16] and the family *Quadriviridae* [17], have been identified *L. biglobosa*. In addition, some *L. biglobosa* strains can be infected by (+) ssRNA viruses belonging to the family *Mitoviridae* [18]. In this article, we detail the full molecular characterization of the first polymycovirus discovered in *L. biglobosa*.

*L. biglobosa* strain W32 was originally isolated from a field of oilseed rape in Canada and maintained in the OREGIN collection of *Leptosphaeria* spp. isolates at the University of Hertfordshire, United Kingdom. *L. biglobosa* was cultured in Petri plates for 7 days at 25°C on yeast glucose agar medium (YGA, 0.5% yeast extract, 2% glucose, 1.5% agar) until conidia were fully developed. Conidia were cultivated in YG broth at 25 °C for 10 days with shaking at 160 rpm to generate mycelia for dsRNA extraction. Total nucleic acid extracts from 0.2 g of dried mycelia were used as a source of dsRNA, which was purified using Advantec D cellulose chromatography [19, 20]. Contaminant DNA and ssRNA were removed by treatment with DNase I and S1 nuclease [20], respectively*.*

Fragmented and primer-ligated dsRNA sequencing (FLDS) was used for viral genome sequencing as described [21, 22]. Raw sequencing data were processed using the FLDS pipeline (available at GitHub, https://github.com/takakiy/FLDS). It total, 2,281,346 reads were generated in the study, and 1,917,447 reads, i.e., approximately 84%, were assembled to build the viral genome sequence. CLC Genomic Workbench version 11.0 was used for *de novo* assembly of contigs >300 nucleotides (nt; other options were default). The nucleotide sequences of the dsRNA segments were confirmed to be complete by manual analysis using CLC Genomic Workbench version 11.0, Genetyx version 14, and Tablet version 1.19.09.03. The depth of coverage for each dsRNA segment ranged from 2.2K to 3.6K. Selected regions were confirmed by Sanger sequencing after amplification by polymerase chain reaction (PCR) with random primers [23] and RNA-ligase-mediated rapid amplification of cDNA ends [24]. The online Multiple Alignment using Fast Fourier Transform (MAFFT v7.511) tool was used for multiple sequence analysis [25]. The neighbour-joining (NJ) method with 1000 bootstrap replicates, as implemented in the Molecular Evolutionary Genetics Analysis (MEGA) 11 software, was used for phylogenetic analysis [26].

Examination by 1.0% agarose gel electrophoresis showed that *L. biglobosa* strain W32 contained several dsRNA elements (Fig. 1A). The results of a Basic Local Alignment Search Tool for Proteins (BLASTp) analysis of the viral ORFs showed that the protein encoded by ORF1 has sequence similarity to the RdRP of Plasmopara viticola lesion associated polymycovirus 1 (PvLAPmV1; 74% identity, 99% coverage, E-value = 0.0), while ORF3 encodes a protein that shares sequence similarity with the PvLAPmV1 methyltransferase (74% identity, 99% coverage, E-value = 0.0). The protein encoded by ORF5 shares sequence similarity with the proline-alanine-serine rich protein (PASrp) of Erysiphe necator associated polymycovirus 1 (EnaPmV1; 50% identity, 89% coverage, E-value = 1e-79). Finally, the proteins encoded by ORF2 and ORF4 have sequence similarities to the corresponding hypothetical proteins of Alternaria alternata polymycovirus 1 (72.13% identity, 100% coverage, E-value = 0.0 and 51.81% identity, 60% coverage, E-value = 1e-15, respectively), a mycovirus identified previously in *Alternaria* sp. FA0703. Collectively, the results of sequence analysis suggested that the five dsRNA segments that were identified constitute the genome of a new polymycovirus, which we have named "Leptosphaeria biglobosa polymycovirus 1" (LbPmV1) (Fig. 1B). A multiple alignment of the nucleotide sequences of dsRNAs 1-5 revealed the presence of the conserved motifs 5'-GGAACUAA—AGUU-UU-3' at the 5' terminus and UUUU at the 3' terminus in all five dsRNAs (Fig. 1C). The complete genome sequence of LbPmV1 has been deposited in the GenBank database under the accession numbers LC859048-LC859052 for the five dsRNA segments.

**Fig. 1:**
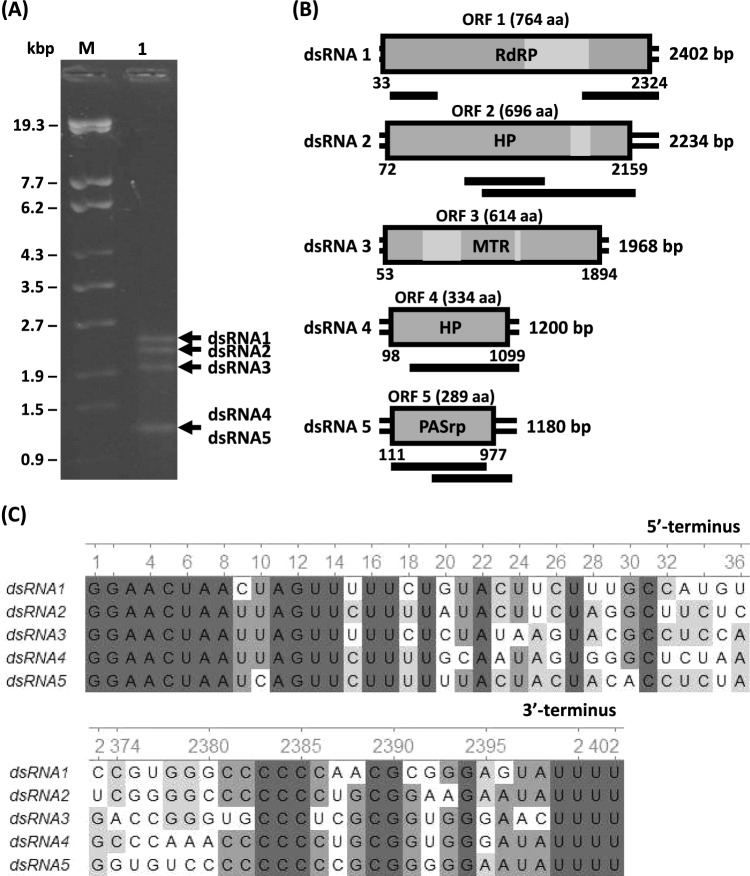
(**A**) Electrophoresis of dsRNA extracted from *L. biglobosa* strain W32 after treatment with DNase I and S1 nuclease. Lane M, DNA molecular weight marker; lane 1, LbPmV1 dsRNA segments. (**B**) Schematic representation of the genome organization of LbPmV1, showing dsRNAs 1-5 with key nucleotides and amino acids indicated. The positions of motifs characteristic of RdRP (PFAM: RdRP_1, aa 412-60), HP (cysteine-rich zinc finger, aa 535-593), and Met (S-adenosylmethionine binding site aa 130-240; catalytic site ARPKFDGHPALLVLKG) are shown as light grey boxes on dsRNA1, dsRNA2, and dsRNA3, respectively. The regions confirmed by Sanger sequencing are shown as black lines under each dsRNA. (**C**) Comparison of the 5′- and 3′-terminal conserved UTRs of the dsRNAs. The black, dark gray, and light gray backgrounds represent regions with at least 100%, 75%, and 50% nucleotide sequence identity, respectively

Using the MUSCLE tool included in MEGA 11 software [26], an NJ phylogenetic tree was constructed to examine the evolutionary links between LbPmV1 and other polymycoviruses. As an outgroup, the RdRP domain of Hadaka virus 1 (HadV1) was used. HadV1 shares a close evolutionary relationship with polymycoviruses [6]. As shown in Fig. 2A, LbPmV1 was found to be most closely related phylogenetically to AaPmV1 and PvLAPmV1, indicating that it is a novel member of the family *Polymycoviridae* and genus *Polymycovirus*. A multiple sequence alignment revealed that the LbPmV1 RdRP contains three conserved motifs (IV, V, and VI) and that motif VI contains a GDNQ motif that is identical to those found in all members of the family *Polymycoviridae* (Fig. 2B). Notably, this motif is typically found in the RdRP of -ssRNA viruses of the order *Mononegavirales*, whereas most dsRNA and +ssRNA viruses have a GDD motif instead [27].

**Fig. 2:**
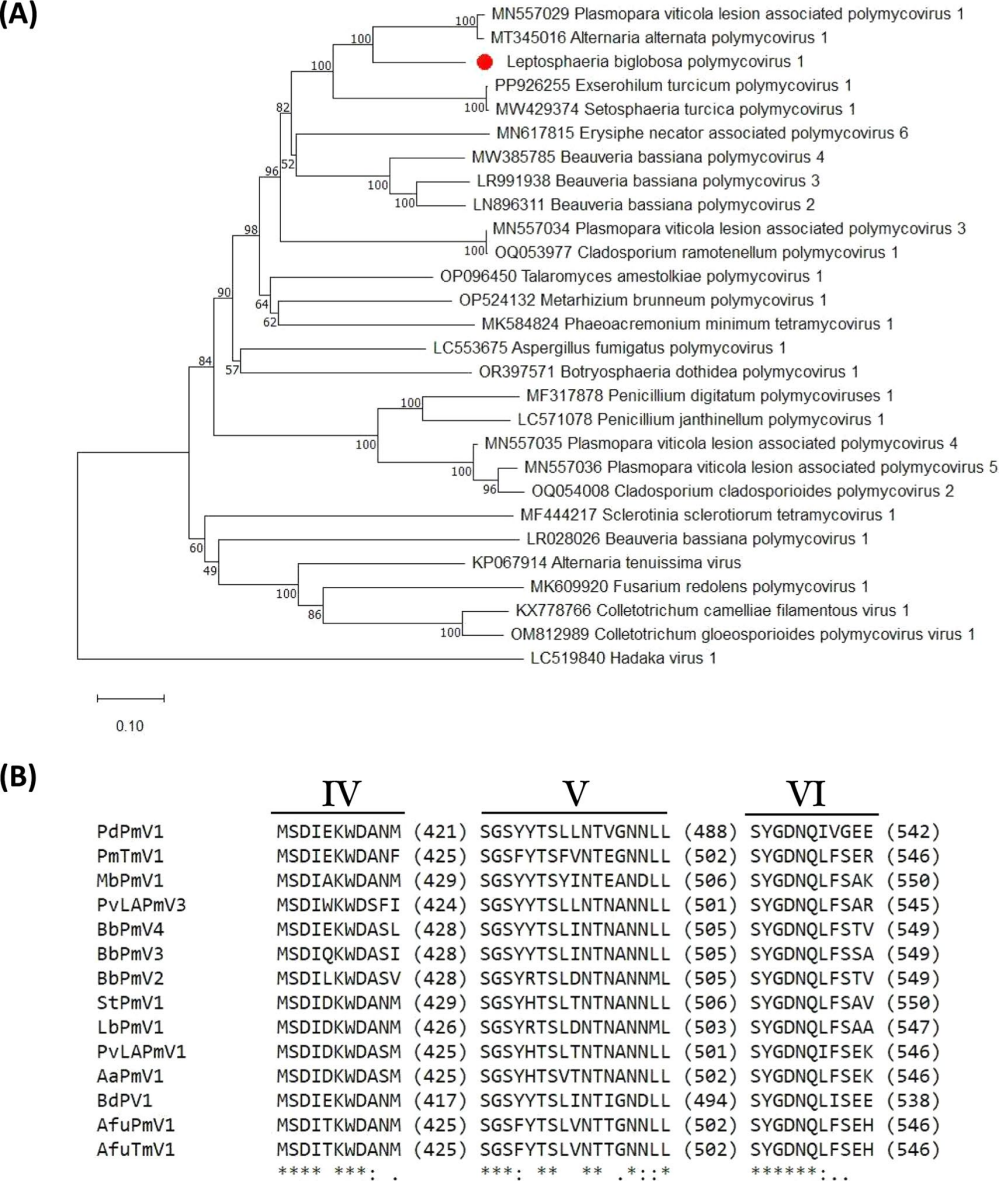
(**A**) Phylogenetic analysis based on an alignment of RdRP amino acid sequences of LbPmV1 and other members of the family *Polymycoviridae*. An NJ tree was constructed in MEGA 11 with 1000 bootstrap replicates. Bootstrap values higher than 50% are shown, and LbPmV1 is indicated by a red circle. (**B**) Amino acid sequence alignment of the RdRP domains of LbPmV1 and other members of the family *Polymycoviridae*, showing conserved RdRP motifs IV to VI

In conclusion, LbPmV1 is the first reported polymycovirus isolated from *L. biglobosa*, and its characterization represents a substantial advancement in our knowledge of the genomic architecture of members of the family *Polymycoviridae* and their evolutionary relationships, particularly in relation to pathogenic fungi affecting economically important crops like oilseed rape. These findings may also guide future investigations into virus-host interactions, as well as potential biocontrol methods.

## Supplementary Information

Below is the link to the electronic supplementary material.

Supplementary file1 (DOCX 22 KB)

Supplementary file2 (DOCX 17 KB)

## Data Availability

The LbPmV1 genome sequence has been deposited in GenBank database under accession numbers LC859048-LC859052.
