# Supplementary material for: Molecular characterization of a polymycovirus in Leptosphaeria biglobosa
Source: Arch Virol. 2025 Mar 6;170(4):66. doi: 10.1007/s00705-025-06253-1 (PMC11885375; doi:10.1007/s00705-025-06253-1)
Supplement: Supplementary file 1 — Supplementary file1 (DOCX 22 KB) [file 705_2025_6253_MOESM1_ESM.docx]

| Virus name | Abbreviation | Accession number |
| --- | --- | --- |
| Alternaria alternata polymycovirus 1 | AaPmV1 | MT345016 |
| Alternaria tenuissima virus | AtRV | KP067914 |
| Aspergillus fumigatus polymycovirus 1 | AfuPmV1 | LC553675 |
| Beauveria bassiana polymycovirus 1 | BbPmV1 | LR028026 |
| Beauveria bassiana polymycovirus 2 | BbPmV2 | LN896311 |
| Beauveria bassiana polymycovirus 3 | BbPmV3 | LR991938 |
| Beauveria bassiana polymycovirus 4 | BbPmV4 | MW385785 |
| Botryosphaeria dothidea polymycovirus 1 | BdPmV1 | OR397571 |
| Cladosporium cladosporioides polymycovirus 2 | CcPV2 | OQ054008 |
| Cladosporium ramotenellum polymycovirus 1 | CrPmV1 | OQ053977 |
| Colletotrichum camelliae filamentous virus 1 | CcFV-1 | KX778766 |
| Colletotrichum gloeosporioides polymycovirus virus 1 | CgPmV1 | OM812989 |
| Erysiphe necator associated polymycovirus 6 | EnaPmV6 | MN617815 |
| Exserohilum turcicum polymycovirus 1 | EtPmV1 | PP926255 |
| Fusarium redolens polymycovirus 1 | FrPmV-1 | MK609920 |
| Metarhizium brunneum polymycovirus 1 | MbPmV1 | OP524132 |
| Penicillium digitatum polymycoviruses 1 | PdPmV1 | MF317878 |
| Penicillium janthinellum polymycovirus 1 | PjPmV1 | LC571078 |
| Phaeoacremonium minimum tetramycovirus 1 | PmTmV1 | MK584824 |
| Plasmopara viticola lesion associated polymycovirus 1 | PvLAPmV1 | MN557029 |
| Plasmopara viticola lesion associated polymycovirus 3 | PvLAPmV3 | MN557034 |
| Plasmopara viticola lesion associated polymycovirus 4 | PvLAPmV4 | MN557035 |
| Plasmopara viticola lesion associated polymycovirus 5 | PvLAPmV5 | MN557036 |
| Sclerotinia sclerotiorum tetramycovirus 1 | SstRV1 | MF444217 |
| Setosphaeria turcica polymycovirus 1 | StPmV1 | MW429374 |
| Talaromyces amestolkiae polymycovirus 1 | TaPmV-1 | OP096450 |
| Hadaka virus 1 | HadV1 | LC519840 |

**Supplementary Table S1.** List of the amino acid sequences of viral RdRps used in the phylogenetic analysis of this study.
