# Supplementary material for: Molecular characterization of a polymycovirus in Leptosphaeria biglobosa
Source: Arch Virol. 2025 Mar 6;170(4):66. doi: 10.1007/s00705-025-06253-1 (PMC11885375; doi:10.1007/s00705-025-06253-1)
Supplement: Supplementary file 2 — Supplementary file2 (DOCX 17 KB) [file 705_2025_6253_MOESM2_ESM.docx]

Strain W32. *Leptosphaeria biglobosa*

Polymycovirus

dsRNA1, 2401nt

GGAACTAACTAGTTTTTCTGTACTTCTTTGCCATGTCGTTTGTCTCTCTGTCCCCTTCGGTTTCTAATCCCATCTTTTCCTCTGCCGCTCCGTCCCCCCTTCCTTCCAACGCCCAGCTCCTTTCGTCACTTCCCGATGTCCTGTTGTACGCCGCTCGTTCGTGCCTCCGTGCAGGCCTGTCCAAGGGGTCGCACCGCTTTTCTGTCGCTCTCCCAGGTGATCCCGCCCCCGTTCCGGTGTTTGTTAACGATGTGTCTGCTGGCCGCGAGCCCCATGTCGCGGTGCAGCATGCTCGTGCTAGCGCTTTCCGGTTGACTGGGAACACCGATGCAGAACGGCTGGAAGCGTTGCGAGCCCACGATGAGCCCAAGCCCAAAGGGACGTCCCGAAAACTTCCGTTTTACCGCTCGTCACACCGTCTCGATTCCTTAGTTGCTGCCTCTTCGTCTCTTGTTCGCCCGTCCGCCGTCGTCTCGCAGTACCAGTTGCCCAAGGCTTTCTCTTTTGGTGGTGGCCCGCCGCCTTCTGACCGTCCCCTCGTCGCGCCTCTTCTTGGAGCCATTGATGCAATGGCCCCTGTCGTCGATGACGCGGCGTACACCAGGTCCATGGAGGACGCGGTGGCCTTGGGTGACTACACGACTCACGACCCCGACACCCTGCCGCCCCGGTTCCTCAACTACCTCCACGAGCGTGTCACGTCCGTAGACGCGGGGACGGCTACCGTCATGGACGCGGCGGCCCTGGCACAACGTCTTCTGTGGGCGCGGTGCGGAGTGGGCGCGACCCCACGCCCATTGTCCGACGCCGCTCCCGAGACTCTCCTCCCCATGCTGAACTCTGGCTCGCCCGGCGAGTATCGATCGTGTGGCGTCCTGGACCGAAGGGATACGCGCCTCGTCGAGGTGATGTCTAAGTCCCTCTTACGGTACGGACGTGCGGGCCGCCTCGCTGCCGCGGGTAAACGGCTCCCTGCGTGGGTCGAAACGACCGTTCAGCCAACTCTCACTTTTGGGAAGGAGGAGCCGAAAGCAGCAAAACGTCTCGCGGATGGCACCGTCGAACCCCCCGTGCCGCGTTTTATTTTCAACCTCAGTCCGATTAACTATGCCCTGTGCGCTTTCCTGCACAGTGATGTGTCGCACTCACTCCAGGAAAACGACCCGACCCACGGCCCAGGCTTCGGCCCAGGCCGCGGTCGATCAGGCAAGTTTCTCAACGTTGTTGAACTCGCGTTTAAGGGGGGCTACACGGTTCCAGCGGGGGAAGAGATGGTCATGTCGGACATAGACAAGTGGGATGCCAACATGCGTGAGGTGTTGCTGGGATACGCGTATGACAACCTTGAAGCTGCGGTCGACAAGTCCGGCCTTTCCCAAGTTGCACTCGCGACCAGGGCTGCCATGTCGAGGGTCTGTAGGCGGCAGCTCCTACACAAGCTGGTTGAACATCCCGCCGGGTACCTTTTGGAGGTCTATGGGACCATGCCGAGTGGCTCGTACCACACCTCGCTCACTAACACCAACGCCAACAATCTTCTCGTCCTCGGCCACATCATCGATCGCGTTTCTACCGAGACACCACTCACTCCCCACTACGCAGCGCAAGAACTCGCTGAGTTGGCTGCGGGGAGGTTGGTGTCGTATGGTGACAATCAACTGTTCTCCGCCGCCCTCTTCAAGCATTTCGGCCTTGCTTACGACGCGCAAAAGCATGCTGAGTTCCTCTCTCGGTTCGGAATGAAGCTCAAGGTCAGTGAGACGGAGGTAACGCGATCCATCGACCGTGTCAGGTTTTGTTCCCGTGCCGTCGTCAGAACGCCGTCCGGACTCCTCATCACGCGCACGCACACGTCTGTGGCCGCGAAACTCGCGGCACGTCCGGAGCATGACGCACTGACAGACAAGCTCTACGTCCGGGCGATAATGGCTGACCATATGGGTACTGACCCCGTCGTGTACGAGATGCTGTCCCAAATTGACCGGCAAATCGAGGTGCCTGTTGACGTCGACACCGTCACACAACGGATCAAGCCGGTCCTGGCTGCGGCTGCCAAGTCCTTGTATGGTTGTGACGAGCAGTGGGCAATAGAGTCGGTGTTGAGTGGGCTCACTACGGCCAGGATCGAGCGTCGGGCTCTGTTGTCTCTTCATACCCCTCGAAAGACCGACACCAGAAGGAATATGAAGGTCGGATCGAGCGTGAACCCGGTTCCGATGTTTGACGGACCTTTAACCCCTGCTGCGTCCTGGGCCAACGAGCAAACGCCGGCGTCGTGGGTGCGCTACCTCCGAGAGACGGGTCAAATGGGGATAATGACTGAATAAAAACGACAAAAATAAATGAAGTACTGTCCTGCGTTTGGGGGCCCCCCGTGGGCCCCCCAACGCGGGAGTATTTT

dsRNA2, 2234 nt

GGAACTAATTAGTTCTTTTATACTTCTAGGCTTCTCCGGTTGGTTTCTGGTCCCCCGGCTCCCCCTGTGTAATGGCAGACCTAACACGTCTGCGCGCCATCCTGCTGGAACGCGGTTCACCTGCTGCCCCGTTAGGATGCGCTATCTACCACCTTACCAGGTCGCCTGTCAAAAGGGTTACCGTCGATGCCGCTCTCGTCGTTGATGTTGTGAAGTGGTTATCATCAGCCCCGGGTGAGTTCTTTGGGGATGTTGTTTCATCCTCAGGGGCTACCCCGGGGGATTTTGGTAGTCTTCTTCACGACATCGACGACAGGAGAGCTGCAGAGACGGCAACCATGGTCAACGCCATGACCATCATTGACGACGAGTGGGATCGGGAGACCAGGGACCAATACTCCACTGCAACCGTTGTCCATGACCGTAACGCCGCTAGGGTTGGTGCCCAGTCCTCCGCCGAGTTCGCTGCCCTTGACGCCCTCACTGCCGCTGGTTTCGCTGGAATCGATGATGCTGTCGGTGCACTCGCTGTTATGCGACGCACCTACAAAAACGATTTCCGCGTTCACGTTAGGCAACTTGCCCGATCGCGCCATGCTGTGACTGCCCGCTTCGTAAGGGATGATGGAACCGTTCGCCTCGTGTTCACGCCGTGGTGTTTATCACGCCGTAATGCACAGGCGTATGCTGCCCTCTGCCTTTCCGACTGGCATCGCGCCATGGTGCAGGACCGCATGAAGGCACGCGCGCGCACGGTGTCGTCGTTCGCCGAGCGCACTTCGCTACAGGCAGTGCGTGACATCATAGCCCCGGCACTTGCACTCCTCACCAGGTACGCTTATGATTCCGCCTCCATGGCTTTCGTGAACGAGTCCGGTGCCGTGGTGCAGAGCGTCGGGTCCAGAGCACCGTACGTCGCTGTTGCCTTCGCGGCGGTGTTTTCCTCCGGCAAGGGTGACGCCGTCATCAATCTCGCGCGCGCTCGTACAGCCGTCGCCAACACCCTCACCAACCCTCTTCCCCCTGACGCGGCCCTCGATTATTTCGCGATGGCCGATTCTCCGTTCGCCGGTCTGGCTCTTTTTTCTCGCGCCCTCGCGGCTTTCCGGACCGAACGTGAGGTGTCGCCATGGGGCGAGAGGCTCAACAAGGGTGCTATGCTCGGCTACGTTGCCAGGGAACGCGACAAGCTAGCTCGCAACACGACCGCGTGCATCAACCGCATCGAGGAAGTCAACGCCCTCATCTCCAGTCTTCCTAACCCGCCACAGTCCCCCCTCCTCGTCGTGGAGTGGGGCGGTCGAGTAGCCGCCCACGCAATTTTAGGGGCTGCCGCGGTTGCGAAGATAGACATCGCTCTTGATGTCGCTGGGTCCGGCGTTGATGTCCCCGGTGTGGATGTCTACGGTGACGATGACGATCCCCCCCATCACTATCAGCTGTACCTCGCGTCCGCAAGGTCTCGCCTTCTCCCTCGCATGCCATCCGTTCCCTACCACGCCGGAACCCCTCTCGTCAGCAAACTCCACGGGATTCTGAACTCAGTCGGTGGACGGGAATCGGGTTTTTCTCTGGTGTATCTCAGCGGAGGCATGTCCCAGCTGGCCGAAACACCCGTGTCCGTGTGCTCCGACTCAAACGCCCGCATGGCCGCACTTGACGGCGCTCGCCTGACCCTTCCCATTGCCTACTACTCGACGGAGGTCTTGCTTCCCCCGGCATGTCACCATGCTGTGTCGCAGGATGCGACGACCTATCTCGAGTCGTGGGGGGCTGGGGAAGATGGGTGCCACCAATGTGAAGCCCACTATCGCGCGTTGTCGTCGGTTGGCGATGCCATGGACCGCGATGGCGTTCGTCTCGTGAAGCCGAGGGCGGTCTTTGCTCACAACGCACTTTTTGGGATTGAGTGGACTGGTCTTGACACGAGCATGGGAGAGTCATTGGAAACCCTGGACGCCGCGGTCGCGTGCAACACTCTCAGGAACTACCAATACGACACTCCGCCTCCCGACCCAATGCCCGGTAAGGACGTTAGCTCTCCGGACCTCATGCAGCTGATGGAAGATCCCGTCCGCCAGGTGCATGCCCTGCTCTCCGGCAATTACGGTGGTCCGATCCGTCCTGAGGACGCCCAGTCAATCGCTGCCAGTGTAGCGTAAAAAGCCTAAAAGTATTCTCCTCCGCTGGGGGGGGCCCCGTTTTTCGGGGCCCCCCCTGCGGAAGAATATTTT

dsRNA3, 1968 nt,

GGAACTAATTAGTTTTTCTCTATAAGTACGCCTCCACGTTTCGCTGCTTAGGATGTTCGCTAGGCAAAGAGATCTTACGGGGCGACGTAGACCCACCCGGGAATTCCCAATACGTCGCACCTCTGACTATTCGCCCCCTAGGCCCAATTCCCTTTCCTCGTTTGCTGTTAGTGTCGCTCGCTCCTCTCGTTCTGGTGCTTCTTCTGTTGTCGGTGACAAGTCCGGTGTCGTTGCGGCTGTTTCTTTACCCCTCACGTTGTTCGAGTATGGCTTTTCCGGTACCGCCCCCCCCGAGCTTGACGTCAACCTCGAGTTCCCCGATGAGGGGGGCTCTCTATTCCTCAACTCGCCTGCAGGGATCCATCTCCGTAACCTCGAGAGGGAACAAACACGCACGGTTCTCCAGTACATTCGCCGGTCCACACGCCTCGCTGGTGCTCGCATTCTTGTCCTCGGTAGTGGCTCTTCTAAGTCAACCGCATCTCTCCTTAACCGCGGCGTTCTCTCCGCCACGTTCGTCGATACCTCCCCGGCCGCTCTCGCGTCACTCCGTAACAACATCAGTGAGACGGGTGTTGATGCGGCCGTGGAAATTGATTACGTGTGCGAAGACGCATGGGAGTTTCTGCGTGGGCTCGACACCGAGCAGTATGATCTCGTCCTAGCGCTTAAGTGTGTCGGTCTCGTTTTGTCTTCTGGTGCTGGCCGCACGACCCGCGAGTTCCTCGATATGGTTGCCGATGTCCTGGCCCCCGATGGAAGCTTTATCACAAACCACCATGCCGCGTTTGCTGAGCCTGAGTGGATCGGAAAGCCCATTGCGGTGGGTATGGACCAACCGTTGTTTGAACTCGCAACAGTCGGCGGGCGGTATGCCGCTGATATCGGATATTCCTGGGACATCTCCCACCCTGATCTCGACCCCGTCGCCCGGTTTTCGTCTCCTGCCGCCCACCATCAGGTCCAAACCTGGCACACTTACCACTTCCGCGCTCGCCATGTCGCTGACGGCCCGAAACTCGGTGTCCCGCACCGTGCTCAACGTACCAAGGCCCCCACAGTGCAGTACGCTCCGGCCCCAGGTGAGTTCGACGAAGTGGCCGACGCCATGATACCTGTCAACAACCGCGGCATCAAACGCATACCTGTGCCCGCGGACGCTGGGTCATTTGACATCCTCCTGGCACGCCCCAAATTTGACGGACATCCTGCTCTCCTGGTACTCAAGGGAACCACCGGCGTCGTCGTTTCGCCCACACGGTCATTCGCCCTACCGCTCCCAGCCGACGTCTCACCACCTCTCATAACCATGTGTGAGGTGGTGGAACCCGCTGAGGGTGGCGTTGTGCTCCCCGTGACTGGTGTGGTACGAATCGGTGACACCCCAACCGATCCCAACGACCTCATGGCCCTCAACAGTGTTGCCCGTGTTCTGGGTATGCTGTCGTCGGCTGGCGTGTTTCCCAGTCTCCCAGGCCTGATGTCGCAACTGCGTGGCAACGATGTGATCCTTCCAGGGGCCGCCGGGCGTGTTCTTACTTTGCCCACCGATGGGGTGAACGTCGTCACGTGCGGGAAGGCCGGTGTGTTCCTTAAGTCGGCGACAGCACACACGATTGACGCGACATCAATCGACATATCCTCGTCCCTGTCTGCCGCGTGCCTAGCTGTCGGTCTCACCGTCCCCCACACCTGTCTGCCCGCGCCCTCTCCTGGCGTGTGGGAGTACTCTCGCGGCCTCTCTGACAACATATGGCGCCCCGTCCGTGAGCGACGAGACAAAACCTACTCTGACACCCCTGGAGCCGTCCTCCACACGTTGCTGGCTTCCCTCGCCGCGCAGGACTCAGGGTTCGTTGGCACCACCGAGGAAATCGCCAAAAAATTTGTGAAGTAGGAGGCGTATTTATAGTGCCCGCCGCGCAGGGCACCTGGCGTTGACCGGGTGCCCTCGCGGTGGGAACTTTT

dsRNA4, 1200 nt

GGAACTAATTAGTTCTTTTGCAATAGTGGGCTCTAACGTATAGTTTCTGTAGGCACAGACCACGTCACTAGACCGTTAGCTAACGACACGACGCACGATGACTTCTGAATGTTCCACCGAGTTTACTCTGCCAGGTGAACCCGGTTCGCGCGGAAACGTGGCCCGATGGGCTCACGCGGTGGCTGAGGAATCCGGGGTTAACCCCGGTGAGCCGCTGCGTCAGCCTGCCCGGTCAGTTTCCGCTCGGTCTGACGTATCAGCTCCCGTGAGCAGGCACGCCGAGGCAACAGGTCCCCCGTCCGCGGCGTCCACTCGCCGCTCCAGTGTCCGCTCGTCCAGGAAGTCGAGCGCGTCAGGGTTTCAACGCCCTGGCTCGTTTCTCGACAACCTCGCGAGCGGAATGGAGCGGTCGGTGACGCGGCGGTCGGCACAGTCCGTCAGGACCGTGTCGAGACCACACTCCACCACCGGCAGGCAATCAGGTGTCGGCTCCAAGCCCGCACCTGAAAGCCTCGCCGACAACCGCCCATCCCCCCCCAACGCCGAGAACACTTCGTCCAGACGTAGCTTGCGTTTAGGGGGGTCCACGGGCGGCTCTCGGCGTTCAACCACGCCACATCACCCTCCCTCCTCCAGCAGGTCGACCCTGGTCTGCCAAGAGGGATGTGCGTGTGGCGCCCCGGCGGTGCACGGAAGTGTGGTTTCTGACACGTCCTCTTCCGTGGTGTCGCCATGGCCAACCCTATCATCCTCCTCGGTACGTGAAGAGGATGCTTCAACACCGGTCGCGTCCCCTGTTGGGATGCGCCGTGGGTCCATGGCGTCAGTACACAACCCCCCCTCCATTCTCCCGGCACCTCTCTCCGTCACCACAACCAGTGTTGACAGGGAAGAGTTTGCGGGGATAATAGCGGACAGGGTTGCGGAGAGGTTGGGAACTGTTCGCCGCGAAGCGGCTGTAATCACTGGTGCGGGCGCAACATCTGCCACCCACCGGCGAGCGAGGTTTACTCGCCCTCCGGAGGGAAGTGGCCGCTTGGCGGCGGCCGCCATGGCATTCGCTGTTGGCCCGGAGAACGTGAGGATAAGGAGCATTCGATAAGAAGCGTGGTCCCACCGTTGGTCCTCAGGACCCCCACTGGAGTTACTCGTCGCGGGGGGGTGCGGTAGCGCCCAAACCCCCCTGCGGTGGGATATTTT

dsRNA5, 1180 nt

GGAACTAATCAGTTCTTTTTTACTACTACACCTCTACGCCTCGCTCCCTAGGAGACAGCCCACGCATCTTCACCCAACACCACACCCGCAACGCCCCTTCCCACCACGCCATGACGCTCAACGACATTATCGGTCGTGACCTTGCCCAGCGCATTGACGTGTTGGGGATCGATGACATCGCCGCGATCATCAAGGCGGCCTCCGTTGGGTTTGGCCCCGCCAAACTCAAGCAGGGGGTTTCCTCCATTGCTCGCGGTGAGGACATCGCTCTCCCTCCCGGCCTGGGCCAGGCCAATCCTCTCACGGTGGTGGCTTGGTCTTTCTGCAGCGACCACGGGCAGTACGCCGATACCTATGGTATCAGCGTGGCCCGTGCCGCTGAACTGAAAGACCAGCTTCGTCGTGACCCTGGTCCGGCCCTTGCGGAGATCCGTGGTGTCGTGTCGGCCCGCCTTGCCTCGCGTGGTTCGCAGCGGCCCGTGGTTGTCGTCACCGACGGCATCCCAGGCGCCGCTACCACCGACCATGCTCCACCCCGCCCCCGGCAGGGCGGTGCTGACCTCAAGAAGGAGATACGCCAAAACGGTGCCCTCTACGGGATGTACAAGTTCGTCGCCGAAGACTCTGGTCGGCCTGGCAACGTCCACTTCCGTGTTCGGCTCGGGTCCGGGCTGTACGCCGTCTTCCCCAACAAAGCTGGGGCCGTCGACGTCGCCCGCCTGTGCCGCGTTCACGGAAGGGGACACCCACCCATCGTCGATCACGTTGCCTTCTGGCGCGACGGCTACCCTCCCCTGTTCGGCGGCGACATCCCCGAGAAGGTCACCTTCGACGGCCGACTTCGTCCCGGTGAGGAACCACCCGCTGACCCCGTTGCCAAGACAAAGACCGATGCCCCTCCCGCTTCTGACCCCACTTCCAGCTCCGTTGACTCCTCCGGGTCACCTGGAAAGGGATCTAGGGGGAGCCCATCGAAGTAGGGCCTTCCCTTCGGACCGCCTGGTGCATACCCCCGCTCCGCCTCGCCCGACAATTGTGCTGTTGCGTAGCTGATAGAGGATCCCCCCCGGGGTGGGAAAGTGCCTAGGTAGAGGTGTAGGTCCGTAATATCCTCCGCGTAGGGGGGGGTACCGGTGCTGTGTGAGAGCACTGGTGTCCCCCCCCCGCGGGGGAATATTTT
